# Assessing protected area vulnerability to climate change in a case study of South African national parks

**DOI:** 10.1111/cobi.13941

**Published:** 2022-06-01

**Authors:** Kevin M. Coldrey, Jane K. Turpie, Guy Midgley, Simon Scheiter, Lee Hannah, Patrick R. Roehrdanz, Wendy B. Foden

**Affiliations:** ^1^ Environmental Policy Research Unit (EPRU) University of Cape Town Rondebosch South Africa; ^2^ Global Change Biology Group, Department of Botany and Zoology University of Stellenbosch Matieland South Africa; ^3^ Senckenberg Biodiversity and Climate Research Centre Frankfurt Germany; ^4^ The Moore Center for Science Conservation International Arlington Virginia USA; ^5^ Cape Research Centre South African National Parks Tokai South Africa

**Keywords:** adaptive capacity, biodiversity, infrastructure, potential impacts, tourism, biodiversidad, capacidad de adaptación, impactos potenciales, infraestructura, turismo, 潜在影响适应能力, 生物多样性, 旅游业, 基础设施

## Abstract

Climate change is challenging the ability of protected areas (PAs) to meet their objectives. To improve PA planning, we developed a framework for assessing PA vulnerability to climate change based on consideration of potential climate change impacts on species and their habitats and resource use. Furthermore, the capacity of PAs to adapt to these climate threats was determined through assessment of PA management effectiveness, adjacent land use, and financial resilience. Users reach a PA‐specific vulnerability score and rank based on scoring of these categories. We applied the framework to South Africa's 19 national parks. Because the 19 parks are managed as a national network, we explored how resources might be best allocated to address climate change. Each park's importance to the network's biodiversity conservation and revenue generation was estimated and used to weight overall vulnerability scores and ranks. Park vulnerability profiles showed distinct combinations of potential impacts of climate change and adaptive capacities; the former had a greater influence on vulnerability. Mapungubwe National Park emerged as the most vulnerable to climate change, despite its relatively high adaptive capacity, largely owing to large projected changes in species and resource use. Table Mountain National Park scored the lowest in overall vulnerability. Climate change vulnerability rankings differed markedly once importance weightings were applied; Kruger National Park was the most vulnerable under both importance scenarios. Climate change vulnerability assessment is fundamental to effective adaptation planning. Our PA assessment tool is the only tool that quantifies PA vulnerability to climate change in a comparative index. It may be used in data‐rich and data‐poor contexts to prioritize resource allocation across PA networks and can be applied from local to global scales.

## Abstract (es)

**Resumen:**

El cambio climático es un gran obstáculo para que las áreas protegidas (AP) logren sus objetivos. Para mejorar la planeación de las AP, desarrollamos un marco de trabajo para evaluar la vulnerabilidad de estas ante el cambio climático con base en la consideración de los impactos potenciales del cambio climático sobre las especies, sus hábitats y los recursos que usan. Además, determinamos la capacidad de las AP para adaptarse a estas amenazas climáticas mediante la valoración de las categorías efectividad de la gestión de las AP, las tierras adyacentes y la resiliencia económica. Los usuarios logran un puntaje y clasificación de vulnerabilidad específicas de la AP con base en las calificaciones de estas categorías. Aplicamos el marco de trabajo a los 19 parques nacionales de Sudáfrica. Ya que todos los parques se manejan como una red nacional, exploramos cómo pueden asignarse de mejor manera los recursos para lidiar con el cambio climático. Se estimaron la importancia de cada parque para la conservación de la biodiversidad de la red y la generación de ganancias. Después usamos las estimaciones para sopesar los puntajes y las clasificaciones generales de vulnerabilidad. Los perfiles de vulnerabilidad de los parques mostraron combinaciones distintivas de impactos potenciales del cambio climático y capacidades de adaptación; los impactos tuvieron una mayor influencia sobre la vulnerabilidad. El Parque Nacional Mapungubwe se ubicó como el más vulnerable ante el cambio climático, a pesar de tener una capacidad de adaptación relativamente alta, principalmente debida a grandes cambios proyectados para el uso de recursos y especies. El Parque Nacional Table Mountain tuvo el puntaje más bajo de vulnerabilidad generalizada. Las clasificaciones de vulnerabilidad al cambio climático difirieron notablemente una vez que se aplicaron los factores de importancia; el Parque Nacional Kruger fue el más vulnerable bajo ambos escenarios de importancia. La evaluación de vulnerabilidad al cambio climático es fundamental para la planeación efectiva de la adaptación. Nuestra herramienta de valoración de las AP es la única que cuantifica la vulnerabilidad de las AP al cambio climático en un índice comparativo. Puede usarse en contextos con muchos o pocos datos para priorizar la asignación de recursos en las redes de AP y puede aplicarse desde la escala local hasta la mundial.

## 利用南非国家公园的案例研究评估保护区对气候变化的脆弱性

【摘要】 气候变化使保护区实现保护目标面临挑战。为了改进保护区规划, 我们开发了一个框架, 在考虑气候变化对物种及其栖息地和资源利用潜在影响的基础上, 评估保护区对气候变化的脆弱性。我们还通过评估保护区管理有效性、邻近的土地使用情况和财政稳健性确定了保护区适应气候威胁的能力。使用者根据对这些类别的打分, 获得针对保护区的脆弱性得分和排名。本研究将该框架应用于南非的19个国家公园。由于这些国家公园是作为一个国家网络来管理的, 我们探讨了如何最好地分配资源以应对气候变化。我们还估计了每个国家公园对网络的生物多样性保护和创收的重要性, 并加权计算了其整体的脆弱性得分和排名。国家公园的脆弱性情况展现出不同气候变化潜在影响和适应能力的组合, 前者对脆弱性的影响更大。马蓬古布韦国家公园尽管适应能力相对较强, 但却最容易受到气候变化影响, 主要是由于其物种和资源使用方面预计会发生巨大变化。桌山国家公园的总体脆弱性得分最低。如果采用重要性加权, 则气候变化脆弱性的排名会出现明显变化;克鲁格国家公园在两种重要性情境下都是最脆弱的。气候变化脆弱性评估是有效的适应性规划的基础。我们的保护区评估工具是目前用比较指数量化保护区对气候变化脆弱性的唯一工具。它适用于数据充足和数据匮乏的情况, 可以确定保护区网络资源分配的优先次序, 并可用于从局部到全球的不同尺度。**【翻译: 胡怡思; 审校: 聂永刚】**

## INTRODUCTION

The nature and extent to which climate change will challenge the effectiveness of protected areas (PAs) is one of the most important conservation questions today (Beale et al., 2013; Hannah et al., 2005). Climate change can compromise the abilities of PAs to meet multiple objectives, including by undermining biodiversity conservation, ecosystem service provision, and economic benefits. Climate change vulnerability assessments (CCVAs) are increasingly popular tools to identify vulnerabilities and guide adaptive management (Small‐Lorenz et al., 2013). To date, most CCVAs of PAs have focused on only 1 aspect of vulnerability—the potential climate change impact on biodiversity (e.g., Belle et al., 2016; Langdon & Lawler, 2015; Perry, 2011). Where the focus has been extended to include nonecological impacts, such as climate change impacts on economic benefits, these have been largely based on expert opinion (e.g., Lemieux et al., 2010; Saunders et al., 2009; Scott & Suffling, 2000).

Understanding the vulnerability of PAs to climate change helps to identify which PAs are at greatest risk, to identify key vulnerabilities of a particular PA or network of PAs, to develop suitable management responses, and to allocate resources for adaptation of a PA system most effectively (Füssel & Klein, 2006; Hannah et al., 2002). A vulnerability assessment is therefore an important step in developing effective conservation adaptation strategies (Glick et al., 2011).

To address the clear need for an integrated approach to assessing vulnerability of PAs, we developed a framework for assessing the relative vulnerabilities of PAs to climate change. Based on assessment of underlying PA characteristics, the framework guides users to assess potential climate change impacts on PA biodiversity and the capacity of PAs to adapt to these threats.

An important consideration when developing an index is the weighting of components, a potentially subjective step that can lead to varying results (Barnett et al., 2008; Eriksen & Kelly, 2007). To avoid this, we applied an equal weighting approach but explored the sensitivity of the results to different index weightings and assumptions. Because vulnerability to climate change is not the only consideration when allocating resources for adaptation between PAs in a network, we included 2 importance measures that can be used to weight the vulnerability results: biodiversity conservation importance and revenue generation importance. These importance indicators are based on PA and network data, thereby reducing subjectivity and contributing to better allocation of management resources.

To test the applicability and utility of the PA CCVA framework, we applied it to South Africa's 19 national parks. The South African national park (SANPark) network is a useful case study because it is data rich and each park has its own unique climate, topography, and water resources, resulting in a diversity of vegetation and species conserved and unique tourist attractions.

## METHODS

**FIGURE 1:**
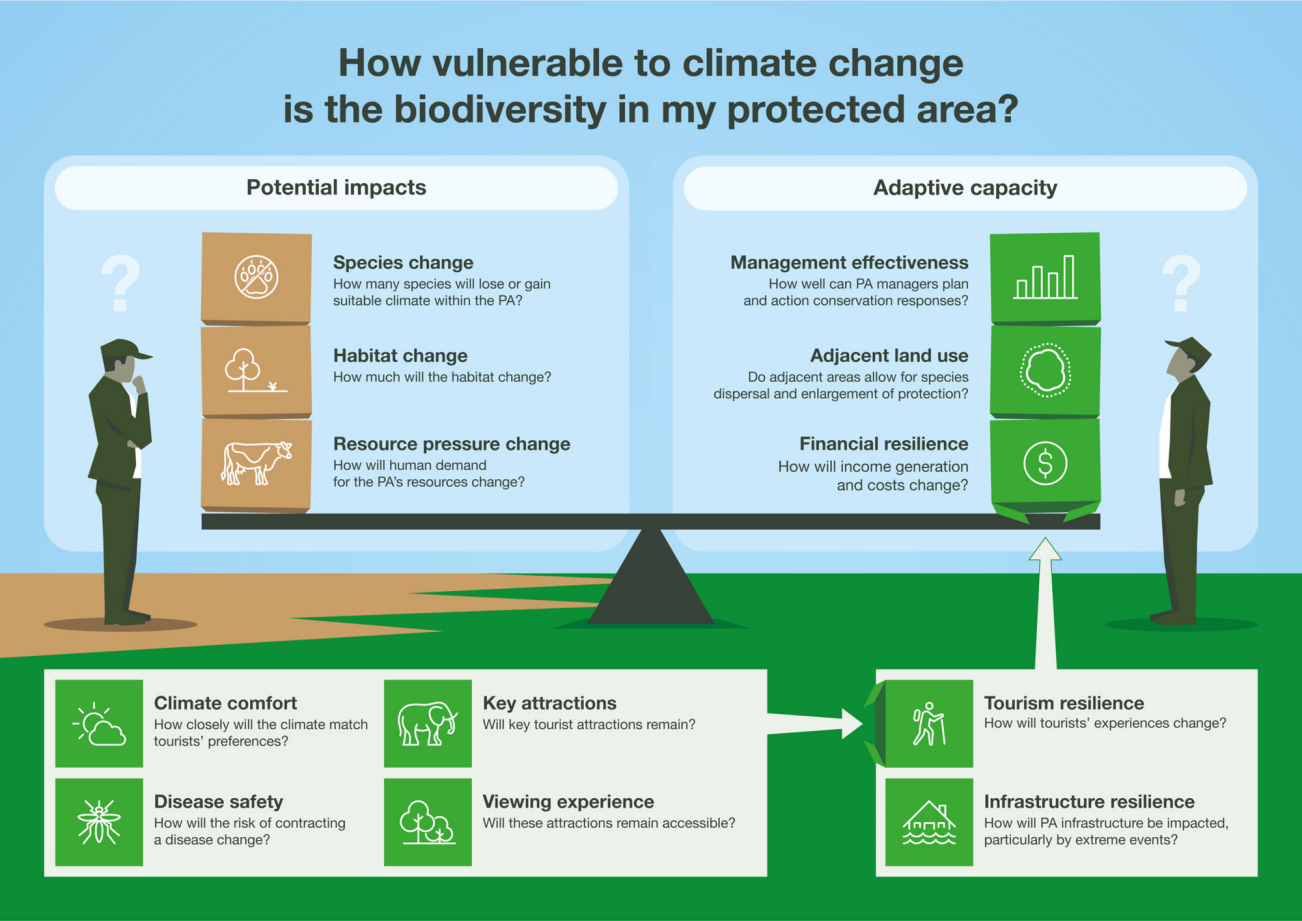
A framework for assessing protected area (PA) vulnerability to biodiversity loss from climate change (left, potential impacts on biodiversity; right, capacity to adapt to impacts). Capacity to adapt includes measures of infrastructure and tourism resilience, the last of which depends on changes in climate comfort, disease safety, key attractions, and viewing experience

A typical CCVA draws on the Intergovernmental Panel on Climate Change's (IPCC, 2014) definition of *vulnerability* that describes vulnerability as a function of sensitivity (degree to which a system is affected, either adversely or beneficially, by climate change) and adaptive capacity (ability of a system to adjust to climate change). To apply this definition to PAs, we used potential biodiversity impacts from climate change as a measure of the PA's sensitivity and compared this with a measure of the PA's adaptive capacity. This measure reflects the risk of climate change negatively affecting PAs’ abilities to meet their conservation mandates (Figure 1; Table 1).

**TABLE 1:** Descriptions of components, categories, and subcategories of the protected area (PA) climate change vulnerability assessment framework

Components, categories, and subcategories	Description
Potential impacts	possible effects of climate change on PA biodiversity
species change	loss of species for which PA is climatically suitable
habitat change	change in habitat composition in PA due to climate change
resource pressure change	change in demand for PA resources by neighboring people due to climate change
Adaptive capacity	capacity of PA managers to cope with the potential impacts of climate change on biodiversity
management effectiveness	success of PA managers at meeting biodiversity conservation objectives
adjacent land use	potential for conservation of species dispersing outside of the PA
financial resilience	capacity of managers to maintain PA income or contain costs despite potential climate‐change‐related impacts
tourism resilience	capacity of PA tourism services to cope with potential climate‐change‐related impacts
climate comfort	extent that altered climatic conditions correspond with tourist preferences
disease safety	change in human disease risk due to climate change
key attractions	change in the prevalence of key PA tourist attractions due to climate change
viewing experience	change in tourists’ capacity to experience key PA tourist attractions due to climate change
infrastructure resilience	capacity of PA infrastructure to withstand extreme events brought on by climate change

The framework guides users in the assessment of 2 components: potential climate change impacts on PA biodiversity and the capacity of PAs to adapt to these threats (Table 1). Categories under potential impacts include changes to species and habitats, and resource pressure from human communities relying on PA resources. Categories under adaptive capacity include management effectiveness, adjacent land use, and financial resilience, which is made up of tourism resilience and infrastructure resilience.

### Potential impacts

PA mandates frequently extend beyond biodiversity conservation to include preservation of cultural heritage and provision of biological resources for sustainable use. Our framework can be modified to accommodate these, but we focused on the risk climate change poses to PA biodiversity mandates due to the pervasiveness of species moving in response to climate change (Pecl et al., 2017). We examined changes in species, habitat, and resource use.

Climate change is a major threat to biodiversity and has already had large negative impacts on individuals, populations, species, communities, and ecosystems (Scheffers et al., 2016). Shifts in species’ ranges as a result of climate change have already been observed (Chen et al., 2011; Mason et al., 2015), and further shifts in range are predicted in response to future climate changes (Araújo et al., 2011; Conradi et al., 2020). To survive, species need to either adapt to their new climate or relocate; otherwise, they face extinction (Moritz & Agudo, 2013; Thomas et al., 2004; Urban, 2015). The vulnerability and expected response of species to climate change has received considerable attention in the scientific community, and a range of data sets are available for species‐level CCVAs (see Foden et al. [2018] for lists of these resources).

Climate change alters habitats and ecosystem structure and functioning through, among other factors, shifts in species’ distributions as they track their climatic niches (Gonzalez et al., 2010), change in plant growth and competition (Poorter & Navas, 2003), changes to the fire regime through increased CO_2_ levels (Bond & Midgley, 2012; Bond et al., 2003), loss of snow cover from increased temperatures (Niittyen et al., 2018), loss of mangroves, wetlands, and floodplains from increasingly variable precipitation (Lovelock & Ellison, 2007; Winter, 2000), and loss of coral reefs from warming oceans and acidification (Burke et al., 2011).

PAs often play an important provisioning role for people near PAs and sometimes living within them (e.g., providing firewood, food, and medicines). However, if resource extraction is unregulated or unsustainably managed, it can lead to degradation and loss of PA resources (Andrade & Rhodes, 2012; de Marques et al., 2016; Guerbois & Fritz, 2017). In low‐income areas with high population densities, people are often more heavily dependent on natural assets and services provided by PAs. They are also typically disproportionately affected by the negative impacts of climate change because they are more dependent on climate‐sensitive natural resources and generally have lower capacity to adapt (Turpie & Visser, 2013). Such peoples’ reliance on PA resources increases during periods of adverse weather (Advani, 2014). CCVAs must therefore consider the extent to which, if improperly managed, climate‐change‐driven increases in resource demand may pose a threat to biodiversity conservation in the PA (van Wilgen & McGeoch, 2015).

### Adaptive capacity

In considering adaptive capacity, we examined management effectiveness, adjacent land use, and financial resilience (i.e., tourism and infrastructure resilience). PAs that are effectively managed have greater chances of adapting to climate change. Management effectiveness evaluations (PAME) are increasingly conducted to assess the effectiveness of PAs in meeting their conservation mandates. A number of PAME tools exist, including the management effectiveness tracking tool (METT) (Stolton et al., 2007) and the rapid assessment and prioritization of PA management (RAPPAM) (Stoll‐Kleemann, 2010).

Land use in areas surrounding a PA can play an important role in allowing species to move to track their suitable climate. Where these areas are largely or totally transformed, they may become impermeable for wildlife dispersal, thereby preventing metapopulation rescue and the dispersal of individuals needed for species range shifts to track changing climatic conditions. Wildlife‐compatible land uses in these zones, including untransformed land and land where custodians accommodate wildlife movement, improve adaptive capacity. Other factors to consider include the type of boundary markers; hard boundaries (e.g., fences) are less permeable than open or soft boundaries (e.g., beacons) for certain species groups, such as large mammals. The extent of human‐affected areas adjacent to PAs and their proximity to adjacent PAs also influence a PA's capacity to support biodiversity under climate change. A decline in revenue or an increase in expenditure will also affect PA managers’ ability to meet conservation mandates. Climate change has the potential to reduce tourism demand, which affects PA revenues, especially for PAs that rely on tourism to finance conservation and operating budgets (Amelung et al., 2007; Scott et al., 2012). Climate change is expected to influence tourism demand directly through a reduction in tourist comfort levels (Coldrey & Turpie, 2020; Fisichelli et al., 2015) and through ecological changes that deter tourists or alter the appeal of a destination. Changes in the distribution of disease‐carrying vectors, such as malaria‐transmitting mosquitos, are predicted. Malaria risk acts as a deterrent to tourism demand because it affects the destination decisions of travellers (Naude & Saayman, 2005; Rossello et al., 2017). The spatial limits of malaria and other diseases are sensitive to climate factors and are predicted to change under a warming world (Caminade et al., 2014; De Souza et al., 2012). However, advances in medicine may mitigate this potential impact. Localized extinction of charismatic species or large decreases in their abundances (Di Minin et al., 2013), particularly due to declining climatic conditions or habitat due to climate change, will affect tourism. Changes in land cover and habitat abundance or density make it more challenging to view charismatic species (Arbieu et al., 2017; Gray & Bond, 2013).

Consumer choices intended to reduce climate change may affect tourism by decreasing demand for recreational long‐distance flights. Alternatively, increasingly uncomfortable weather conditions in tourist origin countries may increase tourism demand. Because we were unable to find reliable data to assess such demand drivers, we have omitted them from this assessment, but recommend their consideration when possible.

Climate change is likely to affect PA infrastructure through changes in the frequency and magnitude of extreme weather events such as flooding, storm surges, wildfires, and extremely strong winds (Davis‐Reddy & Vincent, 2017). Infrastructure damage (e.g., to roads, bridges, and accommodation) can lead to a loss of tourism revenue or increased expenditure on repairs and maintenance, negatively affecting PA finances (Biggs et al., 2014).

### PA vulnerability

Quantifying and scoring the categories and subcategories of the framework allows PA managers to identify those elements contributing to vulnerability at the PA level. There are, however, numerous ways in which they could be quantified, ranked, and categorized. For example, species turnover may provide an indication of both the positive and negative aspects of climate change on species (i.e., a measure that incorporates species gain and loss from a PA), whereas species loss provides an indication of only the negative aspects (i.e., the proportion of species that stand to be lost from the PA). The choice of indicators may be tailored to meet particular needs and to answer questions that may be specific to a particular PA.

### Ranking vulnerability across a PA network

To understand which PAs in a network are most vulnerable, relative vulnerabilities can be calculated and compared. Component scores can be derived as the average of category scores (we scaled the scores to 100 for ease of calculations and interpretation):

$$\begin{array}{ccc} & & \mathrm{potential}\;\mathrm{impact}=\mathrm{average}\;\left(\mathrm{species},\;\mathrm{habitat},\;\mathrm{and}\right.\\ & & \left.\mathrm{resource}\;\mathrm{pressure}\;\mathrm{change}\right) \end{array}$$

$$\begin{array}{ccc} & & \mathrm{adaptive}\;\mathrm{capacity}=\mathrm{average}\;\mathrm{management}\;\mathrm{effectiveness},\\ & & \mathrm{adjacent}\;\mathrm{land}\;\mathrm{use},\;\mathrm{and}\;\mathrm{financial}\;\mathrm{resilience} \end{array}$$

No matter how effective human interventions are to deal with potential effects of climate change on biodiversity, these interventions are unlikely to fully mitigate these impacts. Thus, we propose weighting the adaptive capacity score, for example, by assuming that at most 50% of the potential impacts could be mitigated. Therefore, the overall vulnerability score can be calculated by multiplying the potential impact score by the complement of half the adaptive capacity score:

(1) $$\begin{array}{ccc} \mathrm{Vulnerability} & = & \mathrm{potential}\;\mathrm{impact}\\ & & \times \left[100-\left(\mathrm{adaptive}\;\mathrm{capacity}\times 0.5\right)\right]\%. \end{array}$$

By weighting categories and subcategories in different ways, users can tailor assessments to inform particular conservation questions. For instance, where PA managers are more concerned with direct climate change impacts on biodiversity (such as through species range shifts or habitat change) rather than the indirect impact of higher natural resource use by vulnerable people, additional weighting may be given to the species‐ and habitat‐change categories of the potential impacts’ component. The index may also be weighted to address data quality concerns by weighting good‐quality data higher than poor‐quality data. In our SANParks case study, for example, we tested the sensitivity of vulnerability rankings to 4 different weightings.

### Informing prioritization

Vulnerability rankings alone may not be sufficient for informing adaptation prioritization, given that, in many PA networks, a few PAs are more important with regard to specific functions than others. This could include conserving rare or endemic biodiversity, protecting iconic landscapes, generating revenue, or providing resources. It may therefore be necessary to combine the vulnerability rankings with measures of importance, based on the priorities of the network managers.

### Case study area

South Africa's 19 national parks are managed by SANParks, a state‐owned enterprise. Each park has its own unique combination of climate, topography, and water resources, resulting in a diversity of vegetation and species conserved and unique tourist attractions. Large temperature increases have already been experienced in most parks over the past few decades (van Wilgen et al., 2016), and substantial climate change is predicted for all parks (Appendix S1). The observed temperature changes over the last 20–50 years have, in several instances, already reached those predicted for near‐future scenarios (van Wilgen et al., 2016).

### Indicators of potential impacts and adaptive capacity

Appendix S2 provides details on each of the category and subcategory indicators used in the case study. All future projections and predictions are for the 2050 period based on the same climate data, unless otherwise stated. We were concerned only with the negative impacts of climate change on PAs; therefore, we assigned zeros to positive results.

### Potential impacts

To assess each park's vulnerability to climate change‐driven species loss, we calculated the proportion of the species currently occurring in the park for which future climate is predicted to be unsuitable. This was estimated based on species distribution model outputs that currently overlap with park boundaries carried out for 12,449 species (56 reptiles, 78 amphibians, 463 birds, 170 mammals, and 11,682 plants) by Hannah et al. (2020).

We assessed each park's dissimilarity between current and future biome representation (with a complement of the Jaccard similarity index) by comparing outputs from an adaptive dynamic global vegetation model (aDGVM) developed by Scheiter and Higgins (2009).

We calculated resource pressure change by comparing estimated demand for park resources (fuelwood and bushmeat) relative to estimated sustainable yields. Current resource demand by households living within a 10‐km radius around each park was computed using mapped resource demand (Turpie et al., 2017). Sustainable supply of resources for each park and its 10‐km radius was estimated using yields per vegetation type (Turpie et al., 2017). Future resource demand was estimated by computing the proportion of households at risk of flooding and drought events and was based on the assumption that households increase resource demand during periods of adverse weather.

### Adaptive capacity

We estimated management effectiveness based on each park's latest PAME score, calculated with METT (Stolton et al., 2007).

We assessed adjacent land use as the proportion of untransformed land within a 10‐km radius around each park (GeoTerraImage, 2015).

To assess each park's vulnerability to financial change, we estimated the value of infrastructure at risk from river flooding and coastal storm surges. For river flooding, we used the replacement value of infrastructure within the flood zone of the 1‐in‐100‐year flood return period with a flood hazard model by Sampson et al. (2015). For storm surges, we used the replacement value of infrastructure below the 5‐m contour line calculated from a digital elevation model (Daoudi, 2005).

We estimated potential reduction in tourism demand by summing potential loss in demand as a result of declines in charismatic species (key attractions), decreased tourist comfort levels (climate comfort), decreased game visibility due to greater woody vegetation cover (viewing experience), and increased malaria risk (disease safety).

For changes to key attractions, a list of charismatic species for the region, based on the study by Lindsey et al. (2007) and expert opinion, was used and the same species suitability loss method was applied to determine the potential charismatic species loss for each park.

For changes to climate comfort, estimates of the potential change in tourism demand owing to changes in temperature were used, based on the results obtained by Coldrey and Turpie (2020), where regression analyses were performed on historical occupancy and temperature data for each park to yield a best‐fit model, and future temperature projections were used to predict future occupancy levels.

For changes to viewing experience, the extent of transformation from nonwoody biomes to woodland and forest biomes was calculated using outputs from Scheiter and Higgins’ (2009) aDGVM. For changes to disease safety, the parks projected to be climatically suitable for malaria transmission were assessed using the model developed by Caminade et al. (2014).

### Sensitivity analyses

We compared results obtained using 4 weighting structures to explore the assessments’ sensitivity to different assumptions, as well as how weightings may be useful in informing specific conservation questions. These were carried out with Spearman's rank correlation tests (Appendix S3). The weighting structures were as follows: equal weighting for all potential impacts and adaptive capacity categories and subcategories; higher weighting for factors directly affecting biodiversity (i.e., giving relatively lower weight to resource pressure change); doubling the weight of adaptive capacity scores in the final vulnerability equation, thereby lifting the assumption that efforts by management to adapt to and mitigate the climate threats cannot alleviate all impacts; and selecting the highest value from the 3 potential impact categories (i.e., species change, habitat change, and resource pressure change) for the potential impacts score (This reduced the dilution effect of multiple indicators).

### Informing prioritization

For SANParks, it may be important to prioritize adaptation efforts considering not only the relative vulnerability of PAs to climate change but also their relative contribution to biodiversity conservation and revenue generation within the PA network. The relative contribution results, once normalized (i.e., rescaling the relative contribution results so that the largest value equals 100%), were used to weight the CCVA results for each PA to yield a combined vulnerability and importance ranking.

To estimate relative biodiversity importance, we applied a site endemism index (SEI) (Turpie, 1995) to current distributions of the 12,449 species considered in the species loss analysis (Appendix S4). The index is a measure of relative rarity (Rebelo & Siegfried, 1992) and gives greater value to PAs that contain species that occur in few versus many PAs.

To estimate each park's relative importance for revenue generation, we used the total accommodation units occupied annually for each park (Coldrey & Turpie, 2020).

Depending on management priorities, either biodiversity conservation importance or revenue importance can be combined with vulnerability scores. We normalized the scores for the importance components so that the park with the highest value received a weighting of 100% and subsequent parks were scaled accordingly. These weightings were then multiplied by the vulnerability scores to yield the combined scores:

$$\begin{array}{ccc} \mathrm{combined}\;\mathrm{score} & = & \left(\mathrm{importance}\;\mathrm{score}/\mathrm{highest}\;\mathrm{importance}\;\mathrm{score}\right)\\ & & \times \;\mathrm{vulnerability}\;\mathrm{score} \end{array}$$

## RESULTS

**FIGURE 2:**
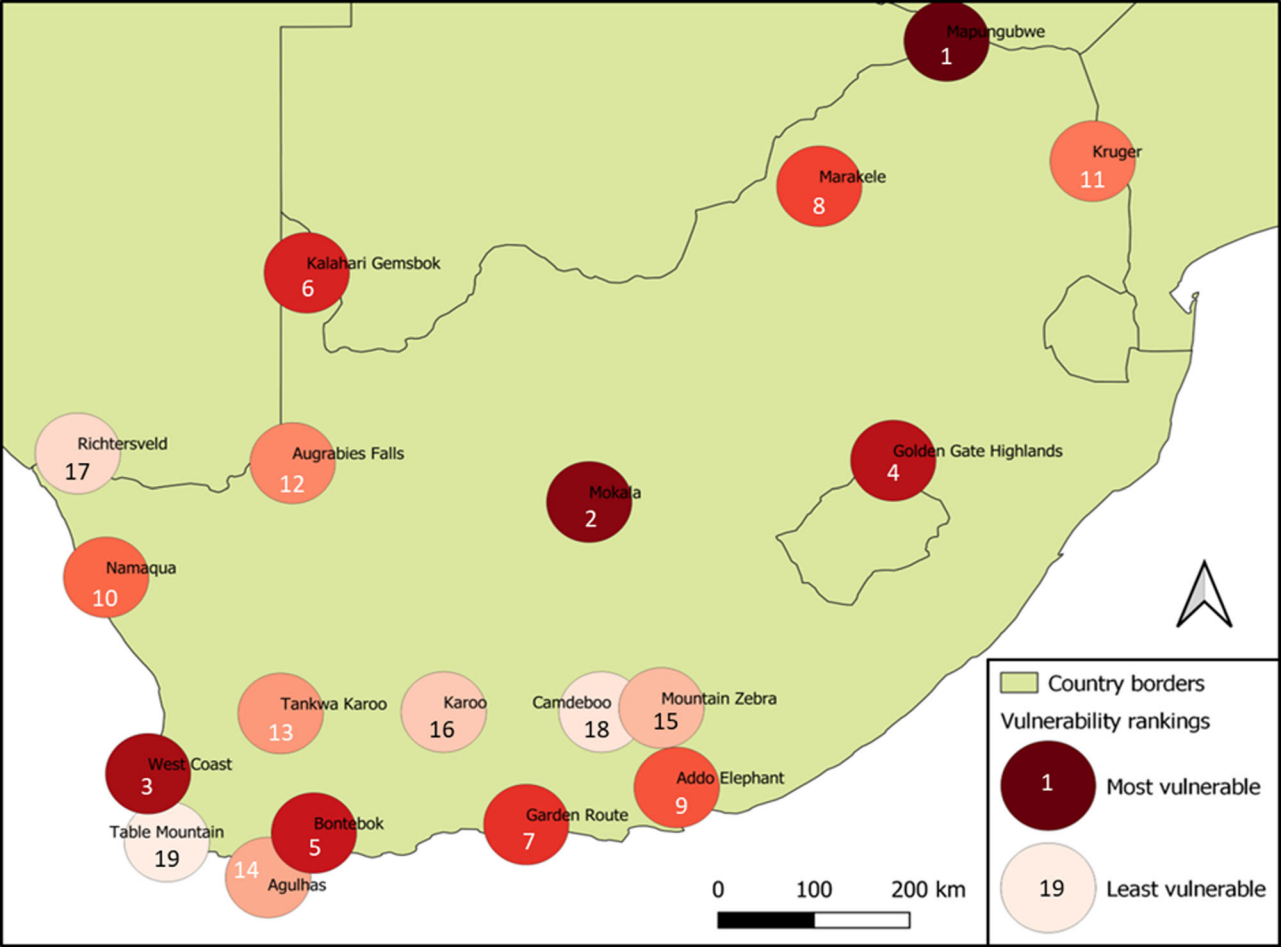
South African national park vulnerability to effects of climate change

**FIGURE 3:**
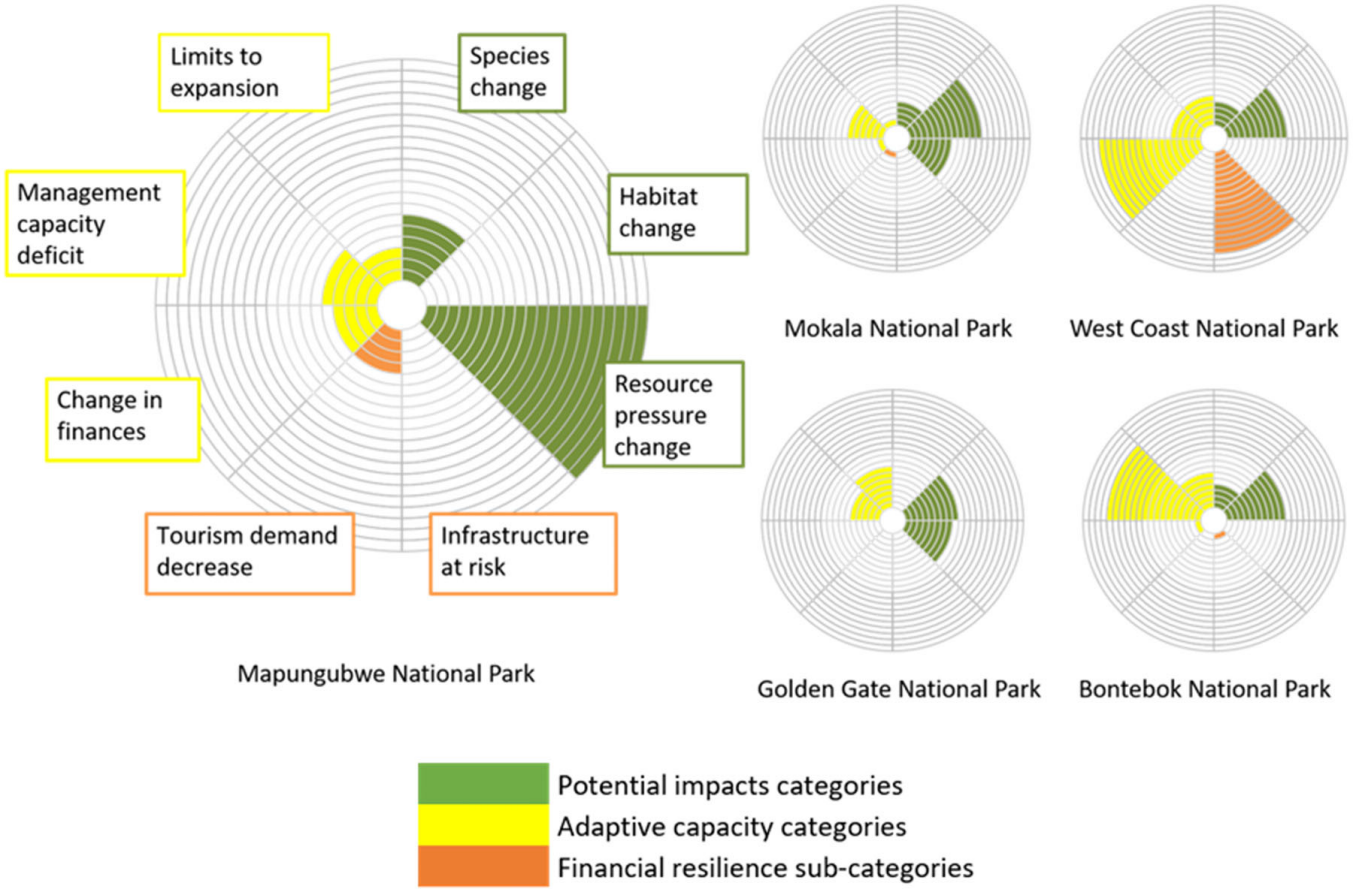
For the 5 national parks in South Africa most vulnerable to effects of climate change, relative scores (each circle represents a score of 5%) for the 3 potential impacts and the complements of the 3 adaptive capacity categories considered. For the adaptive capacity category of financial resilience, scores for contributing subcategories of tourism demand decrease and infrastructure at risk are also shown. The larger the wedge, the greater the vulnerability

The results of the PA CCVA indicated Mapungubwe National Park is the most vulnerable to climate change (Figure 2). This was due to very large potential impacts, driven by high species and resource pressure change, and despite its relatively high adaptive capacity. Table Mountain National Park was least vulnerable (Figure 2), scoring very low on potential impacts and on adaptive capacity. Vulnerability scores followed no geographic or habitat patterns (Figure 3).

Rankings created using different weightings were significantly correlated (Table 2), suggesting that choice of weighting approach did not significantly alter ranking.

**TABLE 2:** Results of spearman's rank correlation used to test alternate approaches to weighting of climate change vulnerability assessment components and categories

Statistics	Factors directly affecting biodiversity	Capacity to adapt to climate change	Maximum potential impact of climate change
Rho	0.975	0.893	0.911
*t*	8.180	18.259	9.080
*p*	0.000	0.000	0.000

**FIGURE 4:**
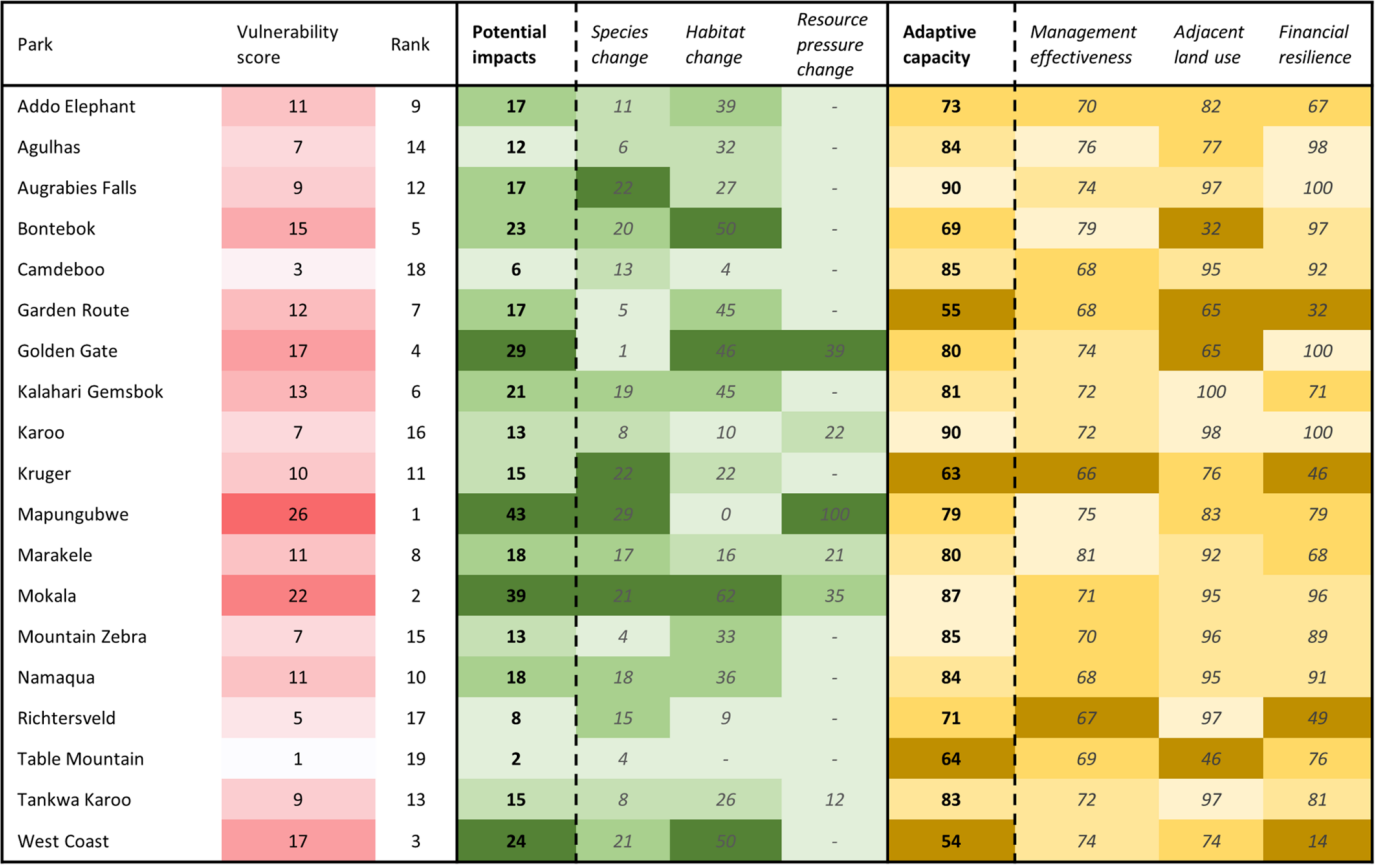
Park climate‐change‐vulnerability assessment scores and ranks; potential impacts and adaptive capacity component scores from which these were calculated; and scores for the 3 categories used to estimate each (bold, climate change vulnerability assessment [CCVA] components; italics, CCVA categories). Vulnerability score color gradient is based on relative rank (most vulnerable park, darkest red). The color gradient for potential impacts and its categories is based on the range of values across the 19 parks (worst performing parks [4th quintile], darkest green; best performing parks [1st quintile], lightest green). The color gradient for adaptive capacity and its categories is based on the range of values across the 19 parks (worst performing parks [4th quintile], darkest yellow; best performing parks [1st quintile], lightest yellow)

Illustrative of the information the PA CCVA framework can provide to PA managers, we considered assessment results for Mapungubwe and West Coast National Parks, both of which ranked among the 5 most vulnerable parks (Figure 4). Mapungubwe's high species‐change score reflected predictions that by 2050 the park would no longer be climatically suitable for 66% of the currently occurring bird species for which models were available (*n* = 171), 43% of the mammals (*n* = 58), 40% of the reptiles (*n* = 10), 43% of the amphibians (*n* = 14), and 24% of the plants (*n* = 1242). In terms of change in resource pressure, 10% of the households within 10 km of Mapungubwe are poor and 100% of the households are at risk of either drought or flood events. Resource demand compared with sustainable supply is already high around the park and given the vulnerability of these households to future climate events, resource pressure was predicted to increase significantly. Adaptive capacity for the park was high, scoring well across all 3 categories (Figures 3 & 4). In terms of financial resilience, Mapungubwe has no infrastructure at risk of river flooding or storm surges, but tourism demand may be affected by predicted changes to tourists’ climate comfort and a potential loss of attractions, in this case charismatic species (Table 3). Just over half of the charismatic species assessed (*n* = 18) were predicted to no longer find the park climatically suitable by 2050.

**TABLE 3:** Financial resilience of parks to climate change and park category and subcategory scores

Park	Financial resilience^a^	Tourism demand decline^b^					Proportion of infrastructure at risk^b^
		total decline	climate discomfort	disease risk	hindrance to viewing	loss of attractions	
Addo Elephant	67	24*	–	20	4	–	7
Agulhas	97†	3	–	–	–	3	–†
Augrabies Falls	100†	–†	–	–	–	–	–†
Bontebok	97	–†	–	–	–	–	3†
Camdeboo	92	2†	–	–	–	2	6†
Garden Route	32*	20	–	20	–	–	46*
Golden Gate	100†	–†	–	–	–	–	–†
Kalahari Gemsbok	71	2†	2	–	–	–	27
Karoo	100†	–†	–	–	–	–	–†
Kruger	46*	14	6	–	3	4	27
Mapungubwe	79	21*	10	–	–	11	–†
Marakele	68	32*	9	20	3	–	–†
Mokala	96	4	–	–	4	–	–†
Mountain Zebra	89	1†	1	–	–	–	10
Namaqua	91	8	–	–	–	8	1†
Richtersveld	49*	3	–	–	–	3	48*
Table Mountain	76	–†	–	–	–	–	24
Tankwa Karoo	81†	7	7	–	–	–	13
West Coast	14*	–†	–	–	–	–	86*

^a^ Range of values across the 19 parks: *, worst performing parks (4th quintile); †, best performing parks (1st quintile).

^b^ Based on the range of values across the 19 parks: *, worst performing parks (4th quintile); †, best performing parks (1st quintile).

West Coast National Park received high scores for both the species‐change and habitat‐change categories of potential impacts (Figures 3 & 4). For species change, 14% of the birds (*n* = 263), 20% of the mammals (*n* = 70), 13% of the reptiles (*n* = 15), 6% of the amphibians (*n* = 18), and 21% of the plants (*n* = 5274) were predicted to no longer find the park climatically suitable by 2050. In terms of habitat change, significant dissimilarity was predicted, reflecting a shift from Fynbos to Succulent Karoo as the region gets hotter and drier. Adaptive capacity was low and largely a result of weak financial resilience (Table 3; Figures 3 & 4). Although tourism demand was not predicted to be affected by climate change by 2050, West Coast National Park has a substantial proportion of its infrastructure (87%) at risk of storm surges linked to sea‐level rise (Table 3), which may necessitate periodic repairs following storm surge events, increasing costs and placing pressure on park finances.

### Informing prioritization

In terms of biodiversity conservation (based on site endemism index) and revenue generation (based on total accommodation units occupied annually), Kruger National Park was ranked the most important park (Table 4). The least important in terms of biodiversity conservation was Augrabies Falls National Park, and least important in terms of revenue generation was West Coast National Park. This resulted in Kruger National Park being ranked first for the combined vulnerability and importance rankings. Table Mountain National Park was ranked last for both (Table 4).

**TABLE 4:** A comparison of park vulnerability to climate change scores and rankings generated under weightings of the relative importance of each park to the network carried out based on biodiversity conservation importance versus revenue generation importance

	Weighted by biodiversity conservation importance			Weighted by revenue generation importance		
Park	site endemism importance scores	vulnerability score^a^	rank	accommodation units occupied annually	vulnerability score^b^	rank
Addo Elephant	7.1	3.9	7	37 295	0.7	5
Agulhas	4.1	1.6	11	3 471	0.0	16
Augrabies Falls	1.6	0.8	17	5 507	0.1	12
Bontebok	3.2	2.6	8	5 241	0.1	10
Camdeboo	3	0.5	18	2 943	0.0	17
Garden Route	7.3	4.6	5	55 000	1.2	3
Golden Gate Highlands	6.6	6.0	3	26 005	0.8	4
Kalahari Gemsbok	1.9	1.3	13	53 790	1.2	2
Karoo	2.3	0.9	15	17 199	0.2	8
Kruger	18.9	10.1	1	554 644	10.1	1
Mapungubwe	4.4	6.1	2	8 211	0.4	6
Marakele	8.6	4.9	4	11 082	0.2	9
Mokala	1.7	2.0	10	8 355	0.3	7
Mountain Zebra	3.4	1.3	12	9 956	0.1	11
Namaqua	3.7	2.1	9	3 909	0.1	13
Richtersveld	4.4	1.2	14	6 082	0.1	15
Table Mountain	6	0.3	19	3 608	0.0	19
Tankwa Karoo	1.8	0.8	16	3 791	0.1	14
West Coast	4.9	4.4	6	534	0.0	18

^a^ Revenue‐generation‐weighted vulnerability score = normalized site endemism index values × unweighted vulnerability score.

^b^ Biodiversity‐conservation‐weighted vulnerability score = normalized accommodation units occupied annually × unweighted vulnerability score.

## DISCUSSION

The PA CCVA framework serves 3 purposes for PA management: it indicates each PA's key vulnerabilities (e.g., Figure 3); it reveals patterns of vulnerabilities (and hence desired responses) across a PA network; and it provides cross‐network rankings of each PA's vulnerability (Figures 2, 3, 4). The information it provides is therefore relevant at scales of individual PAs, regions within a network (which may be grouped, e.g., by biome, geopolitical boundaries, or administrative units), and across entire PA networks. Although our case study explored a national network under a single managing entity, the framework may be used at far greater spatial scales and provides guidance that may foster cooperation across a range of different organizations and administration bodies.

Managers are increasingly faced with the dilemma of deciding how to allocate limited resources to meet PAs’ various and almost always multiple objectives. Glick et al. (2011) explain that such decisions “Will of necessity be based not only on scientific factors, but also social, economic and legal values.” We sought to aid managers in resolving this dilemma by applying an importance weighting to help identify not only the most vulnerable PAs (Figures 2 & 3), but also those that contribute most to meeting the conservation mandate at network level (Table 4). In South Africa, for example, tourism proceeds generated by all 19 national parks are pooled and subsequently reallocated based principally on conservation and operational needs. In this way, a few parks, such as Kruger National Park and Table Mountain National Park, subsidize the conservation of more remote, lower earning parks. The use of the importance weighting combines vulnerability at the park level with how important the park is in either generating revenue for the network pool or for conserving biodiversity. Although our method provides information relevant for managers faced with resource prioritization challenges, results are not prescriptive. They should be used only as a guide, along with stakeholder engagement, experience, and understanding of the specific decision‐making context.

### Caveats, challenges, and the road ahead

At the individual PA level, category and subcategory results (Table 3; Figures 3 & 4) should inform local adaptation strategies. Although measures were taken to reduce the negative effects of indicator dilution, some informative data were lost at the subcategory level that may be useful for PA managers. For example, the species‐change category results for Golden Gate Highlands National Park were relatively benign (1% species suitability loss), but this concealed the modeled projections that 11.5% of amphibian species will have zero remaining suitable climate space by 2050. This information alone may be used to inform adaptation. It is therefore important for PA managers to analyze results in detail to identify where specific vulnerabilities lie. This may help inform development of appropriate and targeted adaptation interventions.

The complex and dynamic natures of ecosystems and climate change make including all potential direct and indirect impacts of climate change in PAs virtually impossible. Even if these were known, finding suitable data or proxies to assess all of them would be extremely unlikely. As a result, selection of assessment components must be based on expert knowledge and further tempered by data at appropriate spatial and temporal scales at which to assess them. The potential biases this introduces may be reduced somewhat through broad expert and literature consultation. In the South African case study, for example, several important potential climate change impacts were omitted, despite climate change impacts being reasonably well understood and data availability relatively good. Climate change impacts are, for example, likely to influence fire regimes, abundance and distributions of non‐native and invasive species, functioning of wetland habitats and their biodiversity, freshwater flows, and functioning of marine habitats and their biodiversity. Also important but omitted because there was no information on sustainable fish yields were the demand and sustainable supply of fish resources. We were also unable to include impacts of changes in land‐use practices in surrounding areas resulting from climate change. These all have the potential to hamper a PA's ability to meet conservation mandates and therefore to affect vulnerability of a PA to climate change.

Despite its frequent use in CCVA, the factors and their definitions chosen to assess sensitivity and adaptive capacity are inherently subjective because they are value based and because factors are restricted to those for which data are available (Fortini & Schubert, 2017; Hinkel, 2011). Response‐based approaches have been explored for species as an alternative (e.g., Fortini & Schubert, 2017), but uncertainties in interacting PA and system responses make this challenging and yet to be explored. Nonetheless, our framework provides scope for updating and expanding assessments as understanding of climate change vulnerability of PAs grows and assessment data increase in scope, volume, quality, and resolution. Any number of additional assessment categories may be added as knowledge and information resources on climate change impacts grow, but its applicability in a broad range of circumstances of information availability remains an asset. To enable assessment updates as knowledge and data resources grow, we urge assessors to clearly record their rationales for inclusion of each component, assessment assumptions, and data sources used.

Our application of the PA CCVA framework to South Africa's national park network made use of detailed park‐level data, which may not be available for other PAs, including in different regions or at different resolutions. If high‐resolution data are unavailable, coarser global data sets could be relied on or, in some instances, proxies could be used. There are growing global data sets on species and vegetation distribution changes under climate change that can be used in most applications. Trade‐offs in the choice of data sets exist and need to be considered carefully. For example, the use of South African census data for the resource pressure component of this study allowed for resource demand to be mapped at high resolution; however, the decision to use this data set came at the expense of ignoring cross‐border pressures. Six of South Africa's national parks share a border with international neighbors, and resource demands by households within 10 km of park boundaries on the other side of the border were not considered in this study, potentially underestimating the pressure of neighboring people on PA resources.

### From vulnerability assessment to conservation action

Tingley et al. (2013) argue that assigning too much weight to climate change in conservation priorities should be avoided due to uncertainties in climate change projections and how ecological systems may respond. Ecological and socioeconomic responses, along with their interactions, greatly compound these. Above all, managers typically face more immediate threats to meeting their conservation mandates. A recent report (IPBES, 2019) shows, however, that climate change will become the dominant threat to biodiversity. Despite uncertainties and challenges, most conservation agencies have begun preparing for climate change and PA managers are increasingly required to develop climate change preparedness and adaptation plans.

Previous studies have identified 3 key considerations for applying CCVA results in PA adaptation. Combinations of each PA's exposure and resilience (i.e., sensitivity and adaptive capacity) may identify it as requiring little intervention (i.e., conditions of either low exposure and high resilience or high exposure and high resilience), traditional conservation (i.e., low exposure and low resilience), or facilitative transition to a new state (i.e., high exposure and low resilience) (Lapola et al., 2020; Magness et al., 2011). Similarly, Schuurman et al. (2020) introduce the resist–adapt–direct (RAD) framework, adding consideration of ecological, societal, and financial feasibility in setting adaptation management objectives. A growing body of literature provides guidance for climate change adaptation (e.g., Mawdsley et al., 2009; Schuurman et al., 2020; Stein & Shaw, 2013; Stein et al., 2014), including specifically for conservation of species (e.g., Mawdsley et al., 2012; Shoo et al., 2013) and PAs (e.g., Gross et al., 2016; Hole et al., 2011).

We emphasize the importance of recognizing and maximizing potential benefits of climate change, such as those in the SANParks’ case studies, which predicted increases in tourist attractiveness due warmer climates, arrival of new climate immigrants, and declines in diseases, such as malaria. To mitigate uncertainty and reduce the potential for maladaptation, adaptation interventions should be assessed using multicriteria analysis, which assists in framing decision problems, to illustrate the performance of alternatives across criteria, explore trade‐offs, formulate a decision, and test decision robustness (Adem Esmail & Geneletti, 2018).

All adaptation responses rely on vulnerability assessment as the foundation from which management interventions can be planned and implemented. The CCVA framework we devised, to our knowledge, is the first to quantify PA vulnerability to climate change via a comparative index.

## Supporting information

Table 1. Projected change in mean annual temperature and total annual precipitation for each park from historic average (1960 – 1990) to 2050 (2040 – 2060), using WorldClim V1.4 and CMIP5 data.
Table 2. Estimated sustainable yields of fuelwood and wild meat (bushmeat) per vegetation type for intact vegetation. Source: Turpie *et al*. (2017).
Table 3. Species identified as ‘key attractions’ in South Africa's national parks.
Table 4. The average cost of replacement per asset type (2016 South African Rands).
Table 5. Weightings applied under the four different weighting structures assessed for the sensitivity analysis.
