# Supplementary material for: Assessing protected area vulnerability to climate change in a case study of South African national parks
Source: Conserv Biol. 2022 Jun 1;36(5):e13941. doi: 10.1111/cobi.13941 (PMC9796953; doi:10.1111/cobi.13941)
Supplement: Supplementary file 1 — Table 1. Projected change in mean annual temperature and total annual precipitation for each park from historic average (1960 – 1990) to 2050 (2040 – 2060), using WorldClim V1.4 and CMIP5 data. Table 2. Estimated sustainable yields of fuelwood and wild meat (bushmeat) per vegetation type for intact vegetation. Source: Turpie et al. (2017). Table 3. Species identified as ‘key attractions’ in South Africa's national parks. Table 4. The average cost of replacement per asset type (2016 South African Rands). Table 5. Weightings applied under the four different weighting structures assessed for the sensitivity analysis. [file COBI-36-0-s001.docx]

**Climate change projections for South Africa’s National Parks (S1)**

Table 1. Projected change in mean annual temperature and total annual precipitation for each park from historic average (1960 – 1990) to 2050 (2040 – 2060), using WorldClim V1.4 and CMIP5 data.

| **Park** | **% change in**  **mean annual temperature** | **% change in**  **total annual rainfall** | **Park** | **% change in**  **mean annual temperature** | **% change in**  **total annual rainfall** |
| --- | --- | --- | --- | --- | --- |
| Addo Elephant | 10.00 | 0.76 | Mapungubwe | 11.62 | 3.20 |
| Agulhas | 9.46 | -9.13 | Marakele | 14.97 | -5.88 |
| Augrabies Falls | 13.47 | -11.84 | Mokala | 16.25 | -4.00 |
| Bontebok | 9.63 | -6.06 | Mountain Zebra | 15.80 | 1.47 |
| Camdeboo | 13.35 | -0.05 | Namaqua | 10.61 | -14.81 |
| Garden Route | 10.96 | -3.14 | Richtersveld | 12.22 | -12.21 |
| Golden Gate | 20.18 | 1.05 | Table Mountain | 9.82 | -11.86 |
| Karoo | 14.69 | -9.28 | Tankwa Karoo | 12.70 | -11.76 |
| Kgalagadi | 14.29 | -5.17 | West Coast | 9.26 | -11.48 |
| Kruger | 10.63 | 5.60 |  |  |  |

**Description of indicators used in the South African National Parks case study (S2)**

**POTENTIAL IMPACTS**

**Species change**

To calculate vulnerability to climate change-driven changes in species composition, we calculated the proportion of species for which the park’s future climate is predicted to be unsuitable. We made use of species distribution model outputs for 56 reptiles, 78 amphibians, 463 birds, 170 mammals and 11 682 plants that currently overlap with park boundaries (Hannah et al., 2020). These were conducted using maxent implemented through the ‘dismo’ package in R. Seven climate variables and 5 soil variables were used as input, considering the timeframe 2060 to 2080 for future projections. Climate data surfaces were drawn from 10 global climate models that had been assessed for skill in Africa and Asia, and skill in reproducing El Niño-Southern Oscillation dynamics. Only species with more than 10 unique occurrence records were modelled. A custom background sampling was used to buffer each species’ range spatially to within 1 000 km of occurrence records used. Geographic ranges were projected for the future within the same buffer area. For absence data generation, both random background sampling (no bias assumed) and target background (occurrences in same family) was used. Trinary maps were developed to capture range uncertainty introduced by threshold choice (Hannah et al., 2020).

**Habitat change**

We assessed dissimilarity between current and future biome representation, making use of the adaptive dynamic global vegetation model (aDGVM; Scheiter & Higgins, 2009). The aDGVM combines well-established routines for simulating photosynthesis, respiration and evapotranspiration with novel models for fire, phenology and allocation within an individual-based framework. The aDGVM simulates the key ecosystem processes responsible for emerging ecosystem structure and function. It is based on cell and organ-level physiological and biogeochemical rates that respond to abiotic environmental drivers and translates these via evolved processes such as resource allocation. This simulates whole plant growth on an annual basis, as well as the way in which this responds to a dynamic environment including disturbance and competition. The aDGVM was run for the African continent at half degree resolution using climate surfaces drawn from 5 global climate models for the period 1970 to 2099. Model results were analysed for the period 2040 to 2060. A dissimilarity measure, the complement of the Jaccard similarity index, was used to estimate the change in biome representation for each park.

The coarse resolution of the aDGVM meant that both Table Mountain and Agulhas national parks fell outside of the aDGVM boundary, so no habitat dissimilarity analysis could be performed using this data source. Instead, habitat dissimilarity was computed using the results of SANParks’ correlative climate envelope model for South African biomes (South African National Parks, 2011). The models differ in that with the climate envelope model, processes important for plant functioning are not simulated. However, it is argued that DGVMs are weak in modelling these processes within the Fynbos biome (Moncrieff et al., 2015), for which both parks are currently fully represented. The same dissimilarity computation was applied.

**Resource pressure change**

We used the magnitude of demand above sustainable supply for fuelwood and bushmeat as the indicator. We calculated resource pressure by comparing estimated demand relative to estimated sustainable yield for park resources. Current fuelwood and bushmeat demand by households living within a 10 km buffer zone around each park was computed using mapped resource demand (Turpie et al., 2017). We assessed sustainable supply of fuelwood and bushmeat for each park and its 10 km buffer zone using estimated yields per vegetation type (Turpie et al., 2017; Table 2). Future resource demand was estimated by computing the proportion of households living within a 10 km buffer zone around each park at risk of flooding and drought events. We assumed that poor households (households with an annual income of less than $ 1 000 (R 19 200), the no-income and low-income categories in South Africa’s 2011 census) within the buffer zone increases resource demand by 400% during times of adverse weather, while the remaining households increase resource demand by 200%. These values are subjective and can be changed by PA managers. Census 2011 data provided population and household income data (CSIR, 2018), the drought hazard map was sourced from Carrão et al. (2016), and the flood hazard map was sourced from Sampson et al. (2015).

Table 2. Estimated sustainable yields of fuelwood and wild meat (bushmeat) per vegetation type for intact vegetation. Source: Turpie *et al*. (2017).

| **Sustainable yields** | | |
| --- | --- | --- |
| **Vegetation types** | **Fuelwood (m^3^/ha/yr)** | **Wild meat (kg/ha/yr)** |
| Grassland | 0 | 1.08 |
| Indigenous forest | 4.26 | 2.15 |
| Thicket / dense bush | 1.39 | 1.61 |
| Woodland / open bush | 0.25 | 1.08 |
| Water seasonal | 0 | 0 |
| Water permanent | 0 | 0 |
| Wetland | 0.28 | 0.54 |

We calculated resource pressure change based on the estimated demand relative to estimated sustainable yield for park resources. If demand was less than sustainable yield, resource pressure was 0%; if demand was twice as much as the sustainable supply, resource pressure was 10%; every multiple above sustainable supply increased resource pressure by 10%. We assumed that demand of ten times the sustainable supply will deplete the resource entirely by 2050.

**ADAPTIVE CAPACITY**

**Management effectiveness**

We estimated management effectiveness based on the latest METT results for each park. The internationally accepted METT was developed by the World Commission for PAs and Worldwide Fund for Nature to provide an over-arching framework for assessing the management effectiveness of PAs world-wide (Stolton et al., 2007). The METT comprises 70 indicators that track, amongst others, the financial capacity (budget), technology, education, information, skills, access to resources, and management capabilities of each park. The latest METT in South Africa is version 4 which was conducted in 2018/19 and is updated biennially. The METT indicators are weighted and aggregated to a score out of 100 where the higher the score the more effectively a park is managed.

**Adjacent land use**

For adjacent land use, the proportion of untransformed land within a 10 km buffer zone was calculated using 2014 land cover data developed by GeoTerraImage (2015).

**Financial resilience**

For changes to park finances, the potential loss in tourism demand owing to climate change and infrastructure at risk of river flooding and coastal storm surges were used as indicators. These were evaluated as follows:

**Tourism resilience**

The potential reduction in tourism demand was estimated by summing the potential loss in demand as a result of a declines in charismatic species (key attractions), decreased tourist comfort levels (climate comfort), decreased game visibility due to greater woody vegetation cover (viewing experience) and increased malaria risk (disease safety). These were evaluated as follows:

**Climate comfort**

For each park, estimates of the potential change in tourism demand owing to changes in temperature were used as the indicator (Coldrey & Turpie, 2020). Regression analyses were performed on historic occupancy and temperature data for each park to yield a best-fit model. Future temperature projections were then used to predict occupancy levels in 2050 and a negative value denoted a loss in comfort and tourism demand. For those parks where the models indicated an increase in tourism demand owing to more favourable temperatures in the future, a value of zero was assigned.

**Disease safety**

For disease safety, the change in range of malaria-carrying mosquito species owing to climate change was used as the indicator, and assessed using the model developed by Caminade et al. (2014). This provided an indication of the parks projected to be climatically suitable for malaria transmission in 2050. It was assumed that increased malaria risk would decrease tourism demand by 20%, a more conservative assumption than the nearly 50% figure indicated by Rossello et al.’s (2017) global study. For those parks where malaria risk declined, a value of zero was assigned.

**Key attractions**

The potential declines in charismatic species in each park owing to climate change was used as an indicator of decline in parks’ ability to attract visitors. A list of charismatic species for the region (n = 18; Table 3), based on the study by Lindsey et al. (2007) and expert opinion, was used and the same species suitability loss methodology applied to determine the potential charismatic species loss for each park. It was assumed that the complete loss of charismatic species would lead to a 20% reduction in tourism demand, a more conservative assumption than the nearly 40% Fig. indicated by Di Minin et al. (2012). The estimated overall percentage loss in charismatic species was therefore multiplied by 20% to estimate the potential change in tourism demand.

Table 3. Species identified as ‘key attractions’ in South Africa’s national parks

| 1 | Cheetah (*Acinonyx jubatus*) | 10 | Oryx (*Oryx gazella*) |
| --- | --- | --- | --- |
| 2 | Black wildebeest (*Connochaetes gnou*) | 11 | Hippopotamus (*Hippopotamus amphibious*) |
| 3 | Blue wildebeest (*Connochaetes taurinus*) | 12 | African Elephant (*Loxodonta africana*) |
| 4 | Nile crocodile (*Crocodylus niloticus*) | 13 | Wild dog (*Lycaon pictus*) |
| 5 | Spotted hyena (*Crocuta crocuta*) | 14 | Lion (*Panthera leo*) |
| 6 | Black rhinocerus (*Diceros bicornis*) | 15 | Leopard (*Panthera pardus*) |
| 7 | Giraffe (*Giraffa Camelopardalis*) | 16 | Warthog (*Phacochoerus africanus*) |
| 8 | Zebra (*Equus quagga*) | 17 | Meerkat (*Suricata suricatta*) |
| 9 | Springbok (*Antidorcas marsupialis*) | 18 | Buffalo (*Syncerus caffer*) |

**Viewing experience**

The abundance and density of woody vegetation have increased markedly over the past few decades (Gray & Bond, 2013; Arbieu et al., 2017) and in many parks now provide a major hindrance to game viewing and bird watching. While the causes of woody vegetation encroachment have been debated, rising atmospheric carbon dioxide concentrations is increasingly accepted as the dominant driver (Wigley et al., 2010; Bond & Midgley, 2012; Buitenwerf et al., 2012; Stevens et al., 2017). To assess vulnerability to declining viewing quality, the extent of transformation from non-woody biomes into woodland and forest biomes, an indication of woody vegetation encroachment, was used as the indicator. This was calculated using the same dynamic global vegetation model used in the habitat component. A threshold of 30% change was used to identify parks where encroachment is significant enough to impact on the viewing experience and therefore tourism demand (Arbieu et al., 2017). It is assumed that woody vegetation encroachment would impact at most 40% of tourism demand, in line with results obtained by Gray and Bond’s (2013) visitor survey conducted in Hluhluwe-iMfolozi Park in South Africa. Furthermore, it is assumed that the severity of woody vegetation encroachment would influence the decline in tourism demand. Therefore, the change in woody vegetation encroachment was multiplied by the maximum 40% change in tourism demand to yield the change in viewing experience impact:

**Infrastructure resilience**

We used the value of infrastructure at risk of river flooding and ocean storm-driven wave surges as an indicator. We calculated the value of infrastructure at risk of river flooding, the replacement value of infrastructure lying within the flood zone of the 1-in-100-year flood return period. The flood hazard model used makes use of localised flood frequency analysis (vs. hydrological models) to produce 90 m spatial resolution return period flood maps globally (Sampson et al., 2015). The flood model output was then overlaid with park infrastructure data obtained from Tracks4Africa (received June 2017) to develop a list of park assets at risk of flooding.

We calculated the value of infrastructure at risk of storm surges as the replacement value of infrastructure below the 5m contour line, using a digital elevation model (Daoudi, 2005). The 5m contour line has been used in studies to indicate the upper limit of the damage zone for climate impacts based on a 1-in-50-year wave return period in the year 2100 (Rautenbach, 2015).

South African National Parks’ (SANParks’) asset registers for each park do not include replacement values, so to estimate these an average cost of replacement per asset type was developed based on the most recently acquired assets (Table 4). To determine the replacement value of each park’s total infrastructure, an average age of 10 years for existing infrastructure was used. We assumed that the cost of replacement of infrastructure has grown by 6% per year (in line with historical inflation rates) to yield the present value of replacing all infrastructure per park.

We calculated infrastructure resilience as the complement of the replacement value of infrastructure at risk as a proportion of total infrastructure.

Table 4. The average cost of replacement per asset type (2016 South African Rands).

| Bridge | R 5,00 m | Picnic spot | R 0,09 m |
| --- | --- | --- | --- |
| Entrance gate | R 0,50 m | Ablutions | R 0,95 m |
| Hide | R 0,05 m | Lodge | R 5,00 m |
| Safari tent | R 0,10 m | Hut | R 0,50 m |
| Chalet | R 1,00 m | Fuel garage | R 5,00 m |
| Staff house | R 0,30 m | Restaurant | R 15,00 m |
| Reception | R 0,25 m | Shop | R 5,00 m |

**Weightings applied in the sensitivity analysis (S3)**

Component scores can be computed by applying weightings to their category scores:

*Potential Impact = x*species + y*habitat + z*resource pressure change*

*Adaptive Capacity = a*management effectiveness + b*adjacent land use + c*financial resilience*

where x+y+z=100 and a+b+c=100

An overall vulnerability score can be computed by multiplying the potential impact score by the complement of half the adaptive capacity score.

*Vulnerability = Potential Impact * (100 – (Adaptive Capacity/2)) %*

The following four different weighting structures were assessed using Spearman’s Rank Correlation tests (Table 5):

1. Equal weighting for all potential impacts and adaptive capacity categories:
2. Emphasizing the direct climate impacts on biodiversity by reducing the weight applied to the resource pressure change category of potential impacts:
3. Lifting the assumption that efforts by management to adapt to and mitigate the climate threats cannot alleviate all impacts by not halving the adaptive capacity score in the final vulnerability equation; and
4. Using the highest score out of the species change, habitat change or resource pressure change categories for the potential impacts score which reduces dilution effect of multiple indicators for potential impacts.

Table 5. Weightings applied under the four different weighting structures assessed for the sensitivity analysis

| **Weighting structure** | **Potential impacts** | **Species change (x)** | **Habitat change (y)** | **Resource pressure change (z)** | **Adaptive capacity** | **Management effectiveness (a)** | **Adjacent land use (b)** | **Financial resilience (c)** |
| --- | --- | --- | --- | --- | --- | --- | --- | --- |
| 1 | 100 | 33.3 | 33.3 | 33.3 | 50 | 33.3 | 33.3 | 33.3 |
| 2 | 100 | 40 | 40 | 20 | 50 | 33.3 | 33.3 | 33.3 |
| 3 | 100 | 33.3 | 33.3 | 33.3 | 100 | 33.3 | 33.3 | 33.3 |
| 4 | 100 | Max (x, y, z) | | | 50 | 33.3 | 33.3 | 33.3 |

**Description of the computation of the Site Endemism Index (S4)**

The Site Endemism Index (SEI) is calculated using the formula:

SEI = k/a_i_

where k = the total number of PAs, and a_i_ = number of PAs in which the ith species occurs.

The SEI has been modified by applying weights to individual species based on their proportion to their taxonomic group and not to the total number of species assessed. This reduces the dilution effect that the more numerous plant species would have on the other taxonomic groups considered. The SEI scores for each species were multiplied by the proportion each species contributes to their relevant taxonomic group, based on the total number of species assessed in each group:

SEI * (1/X),

where X = the number of species assessed within the amphibian, reptile, mammal, bird and plant taxonomic groups. The SEI scores for each species currently overlapping with a park were summed to yield each park’s weighted SEI score.
